# Multifaceted Functions of Protein Kinase D in Pathological Processes and Human Diseases

**DOI:** 10.3390/biom11030483

**Published:** 2021-03-23

**Authors:** Xuejing Zhang, Jaclyn Connelly, Yapeng Chao, Qiming Jane Wang

**Affiliations:** Department of Pharmacology and Chemical Biology, University of Pittsburgh, Pittsburgh, PA 15261, USA; xuz20@pitt.edu (X.Z.); jac444@pitt.edu (J.C.); yac63@pitt.edu (Y.C.)

**Keywords:** protein kinase D, diseases, cancer, cardiovascular diseases, CNS disorders, metabolic diseases, inflammation, immune dysregulation

## Abstract

Protein kinase D (PKD) is a family of serine/threonine protein kinases operating in the signaling network of the second messenger diacylglycerol. The three family members, PKD1, PKD2, and PKD3, are activated by a variety of extracellular stimuli and transduce cell signals affecting many aspects of basic cell functions including secretion, migration, proliferation, survival, angiogenesis, and immune response. Dysregulation of PKD in expression and activity has been detected in many human diseases. Further loss- or gain-of-function studies at cellular levels and in animal models provide strong support for crucial roles of PKD in many pathological conditions, including cancer, metabolic disorders, cardiac diseases, central nervous system disorders, inflammatory diseases, and immune dysregulation. Complexity in enzymatic regulation and function is evident as PKD isoforms may act differently in different biological systems and disease models, and understanding the molecular mechanisms underlying these differences and their biological significance in vivo is essential for the development of safer and more effective PKD-targeted therapies. In this review, to provide a global understanding of PKD function, we present an overview of the PKD family in several major human diseases with more focus on cancer-associated biological processes.

## 1. Introduction

Protein kinase D (PKD) was discovered near the turn of the second millennium, with PKD1 first reported in 1994 [1,2], followed by PKD3 in 1999 [3] and PKD2 in 2001 [4]. Due to the presence of a diacylglycerol (DAG)-binding C1 domain in its structure, PKD was initially classified as an atypical protein kinase C (PKC) and given the name PKCµ for human PKD1 and PKCν for PKD3. Later, recognizing the similarity of its catalytic domain to Ca^2+^/calmodulin-dependent protein kinases (CAMKs), PKD was reclassified into the CAMK group in the human kinome. Interestingly, PKD not only binds DAG but also is activated by PKC through direct phosphorylation, a unique feature allowing PKD to integrate signal inputs from both DAG and PKC. 

In the past two decades, extensive progress has been made towards the understanding of PKD structure, regulation, function, and signaling mechanisms. PKD has now emerged as a key signaling node in the DAG network, activated by a variety of cellular stimuli including growth factors, G protein-coupled receptor (GPCR) agonists, hormones, bioactive peptides, cellular stresses, and cytokines/chemokines, and coordinately regulates various downstream cellular processes, such as proliferation, survival, motility, secretion, and gene expression. PKD resides or can be mobilized to different subcellular locations, including the plasma membrane, Golgi, mitochondria, and nucleus, to carry out unique functions. Coinciding with its important roles in normal biology, dysregulation of PKD has detrimental impacts and associates with a variety of pathological conditions and diseases such as cancer, cardiac diseases, metabolic disorders, inflammatory diseases, neuronal dysfunctions, and immune dysregulation. 

In this review, we outline past findings and new studies on PKD in various pathological conditions and diseases. Due to the space limitation, we only highlight the key findings that link PKD to specific diseases and their implications in therapy. In areas that have been more extensively studied, such as cancer, the discussion focuses on the diverse and often interconnected roles of PKD underlying the pathogenic processes of the disease.

## 2. PKD Structure and Regulation

### 2.1. Structure, Isoforms, and Expression/Tissue Distribution

**Figure 1:**
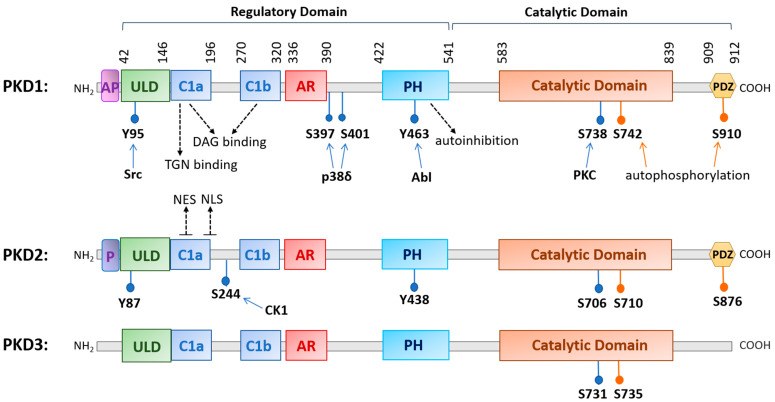
A diagram illustrating the conserved structural domains and major phosphorylation sites in human protein kinase D (PKD) isoforms. The structure of PKD contains a newly identified ubiquitin-like domain (ULD) for dimerization, a C1 domain (Cla and Clb) that binds diacylglycerol, a pleckstrin homology (PH) domain for autoinhibition, a catalytic domain for substrate phosphorylation, and a PDZ domain in PKD1 and PKD2 for protein interactions. Other domains with less known functions are the acidic amino-acid-rich region (AR) and an alanine–proline-rich region (AP) for PKD1 and a proline-rich region (P) for PKD2. Major phosphorylation sites and the upstream kinases that confer the phosphorylation are indicated as well as the nuclear export signal (NES) and nuclear localization signal (NLS) for PKD2. Abbreviations: trans-Golgi network (TGN), Abelson murine leukemia viral oncogene homolog 1 (Abl), casein kinase 1 (CK1).

The family of PKD is evolutionarily highly conserved with three isoforms (PKD1, PKD2, and PKD3) identified in mammals. The three PKDs are similar in sizes (912, 878, and 890 amino acids (aa) for human PKD1, -2, and -3, respectively). PKD1 and PKD2 are most homologous and may originate from a common ancestor [5]. PKDs are widely distributed in the body, although their relative expression varies with different organs, with PKD1 and PKD2 being more prevalent in the lung, brain, kidney, heart, smooth muscle, pancreas, and prostate, and with PKD3 being more ubiquitous [1,2,3,4,6]. The conserved structure of PKD contains a N-terminal regulatory region that mainly comprises a C1 domain and a pleckstrin homology (PH) domain, followed by a C-terminal catalytic domain. The C1 domain constitutes two cysteine-rich Zn-finger-like motifs, Cla and Clb, that bind DAG and phorbol esters, the pharmacological analogs of DAG, with high affinity and regulate PKD localization to the nucleus, Golgi, and plasma membrane [7,8]. The PH domain exerts an autoinhibitory function on the catalytic domain to keep the kinase inactive at basal state [9,10]. Additionally, the regulatory domain contains an alanine–proline rich (AP) region at the N-terminus for PKD1 and a proline-rich (P) region for PKD2, and an acidic amino-acid-rich region (AD) between Clb and PH domains, and the functions of these domains remain obscure [11]. Recently, a ubiquitin-like domain (ULD) shared by all three PKD isoforms was identified at the N-terminus following the AP or P region. Based on the X-ray crystal structure of the ULD-C1a domain in the *C. elegans* PKD homolog DKF-1, ULD may act to initiate PKD dimerization at the membrane for trans-autophosphorylation at the activation loop in response to increased DAG concentration, leading to PKD activation possibly independent of PKC [5,12]. This domain appears to be conserved in all three human PKD isoforms [5,12]. The structure of PKD1 and PKD2 also contains a C-terminal PDZ domain that is thought to facilitate protein substrate recognition [7]. Within the PDZ domain, there is an autophosphorylation site (S910 for PKD1, S876 for PKD2), which is commonly used as a measure for PKD activation status, although its phosphorylation likely also plays a functional role [7,13,14] (see Figure 1 for a schematic diagram of human PKD1, -2, and -3). 

### 2.2. Mechanisms of Regulation

PKD can be activated downstream of GPCRs or receptor tyrosine kinases (RTKs) by a variety of stimuli such as hormones, growth factors, neuropeptides, lipids, and cellular stresses [7]. In a canonical activation pathway, following receptor stimulation, phospholipase Cs (PLCs) are activated to hydrolyze phosphatidylinositol 4,5-biphosphate (PIP2) to generate inositol 1,4,5-trisphosphate (IP3) and DAG. IP3 mobilizes internal calcium and DAG along with calcium (for cPKC) binds and anchors classic or novel protein kinases C (c/nPKC) to the plasma membrane and triggers their activation. DAG also recruits cytosolic PKD to the plasma membrane by binding to its C1 domain; this process may induce a conformational change that allows PKC to colocalize with PKD at the plasma membrane to transphosphorylate a conserved serine residue (Ser^738^ for PKD1, Ser^706^ for PKD2, Ser^731^ for PKD3) in the activation loop of PKD, leading to the autophosphorylation of an adjacent serine residue (Ser^742^ for PKD1, Ser^710^ for PKD2, and Ser^735^ for PKD3) and relief of autoinhibition by the PH domain for full activation of the kinase [15,16,17]. Given the lack of crystal structure of PKD, there remain many questions regarding the exact sequence of events occurring during the activation of PKD by DAG and PKC, particularly in light of the discovery of a N-terminal ULD domain (see detailed discussion in ref. [5]).

**Figure 2:**
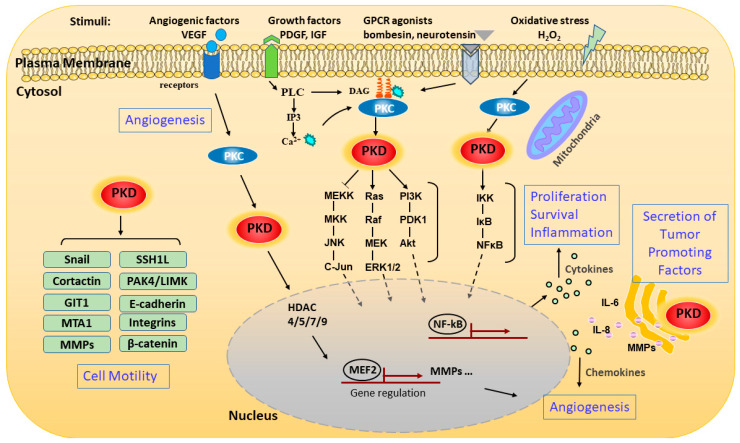
Schematic representation of signaling pathways and pathological processes regulated by PKD. The schematic representation shows the pathways that activate PKD and the various downstream signaling events and functions modulated by the kinase. PKD can be activated through stimulating various membrane receptors such as G-protein-coupled receptors (GPCRs) and growth factor receptors. The extracellular stimuli activate phospholipase C (PLC), which catalyzes the formation of diacylglycerol (DAG). DAG modulates PKD activation by binding and recruiting it to the cell membrane for activation by protein kinase C (PKC). PKD can also be activated on the outer mitochondrial membrane by oxidative stress through binding to DAG and PKC. Activated PKD is rapidly translocated from the plasma membrane to the cytosol and then to the nucleus, where it regulates a set of transcription factors in the nucleus. Activated PKD regulates a battery of pathological processes including cell proliferation, survival, migration, invasion, gene transcription, inflammation, angiogenesis, and secretion of tumor-associated factors through several major signaling pathways. Abbreviations: matrix metalloproteinase (MMP), vascular endothelial growth factor (VEGF), platelet-derived growth factor (PDGF), insulin-like growth factor (IGF), metastasis-associated 1 (MTA1), slingshot-1L (SSH1L), GPCR kinase-interacting protein 1 (GIT1).

PKD can be activated at multiple subcellular locations and can also be mobilized to different cellular compartments to carry out unique functions at each site (Figure 2). PKD was first identified as a trans-Golgi network (TGN)-resident enzyme [18]; a series of landmark studies by the Malhotra group shows that PKD plays a critical role in regulating the fission of transport carriers from TGN to the cell surface [19,20,21]. As with its activation at the plasma membrane, PKD is recruited by DAG to the TGN [22]. An intact C1a domain and the catalytic activity of PKD are both required for its binding and regulation of vesicle trafficking at the TGN [20,23]. The regulation of the TGN by PKD is essential for many important secretory processes, and the best known is the regulation of insulin secretion from pancreatic β cells [24] A well characterized substrate of PKD at the TGN is phosphatidylinositol-4 kinase IIIβ (PI4KIIIβ). Phosphorylation and activation of PI4KIIIβ is critical for protein transport at the TGN [25].

Following its activation at the plasma membrane, activated PKD can shuttle in and out of the nucleus to regulate the activity and availability of several transcription factors. Nuclear localization of PKD is regulated by more than one region of the domain structure, and the precise export and import mechanisms for each isoform may vary with the specific isoform and cellular context. In unstimulated cells, PKD1 and PKD2 exist primarily in the cytoplasm, while PKD3 is present in both the nucleus and cytoplasm as a result of continuous shuttling [26]. In earlier studies, it was shown that the Clb motif controls the nuclear import of PKD1, with evidence supporting the use of a nonclassical nuclear localization signal (NLS). The nuclear export of PKD1 is regulated by the PH domain in a Crm-dependent manner [27]. A recent study identified a complex formed between the catalytic domain of PKD1 and Hsp20, which is essential for PKD1 nuclear translocation and downstream signaling events leading to cardiac hypertrophy [28]. This supports a potential complex and cell context-dependent regulation of PKD1 nuclear localization. For PKD2, an isoform that has been more extensively studied in this aspect, the C-terminus of C1a and the linker region between Cla and Clb contains a putative bipartite NLS (key residues 192RKRR195), which is critical for the nuclear import of PKD2, while Cla contains a functional nuclear export signal (NES) that is required for the nuclear export of PKD2 [29] (Figure 1). Interestingly, the nuclear location of PKD2 can be regulated by a casein kinase 1-mediated S244 phosphorylation in the C1a/C1b linker region of PKD2 [30]. For the nuclear import of PKD3, it appears that the catalytic activity of the kinase is essential, implying the involvement of other import partners, and the export is similarly dependent on Crm1 [26,31]. 

PKD can also be activated in response to an increase in mitochondrial or cellular reactive oxygen species (ROS). Work by the Toker and Storz groups shows that increased ROS induces several tyrosine phosphorylations in PKD1 (Tyr^95^, Tyr^432^, Tyr^463^, and Tyr^502^) [32,33,34]. Furthermore, Abelson murine leukemia viral oncogene homolog 1 (Abl) directly phosphorylates PKD at Tyr^463^ in the PH domain to initiate a conformational change which allows PKD1 to translocate to the mitochondrial membrane through binding to DAG, which is generated by activated phospholipase D1 downstream of mitochondrial ROS [35]. Phosphorylation of PKD1 at Tyr^95^ by proto-oncogene tyrosine kinase Src then allows PKCδ to bind and fully activate PKD1 via activation loop phosphorylation. PKD1 then induces the activation of the Nuclear Factor-κB (NF-κB) signaling pathway to promote cell survival and detoxification of mitochondrial ROS via induction of manganese-dependent superoxide dismutase (MnSOD) [33,34,36]. In recent years, isoform- and cell context-specific regulation of ROS-induced PKD activation has been observed along with downstream signaling events. Detailed discussions on this topic can be found in three reviews (see references [13,37,38]). 

## 3. PKD in Pathological Processes and Human Diseases

**Figure 3:**
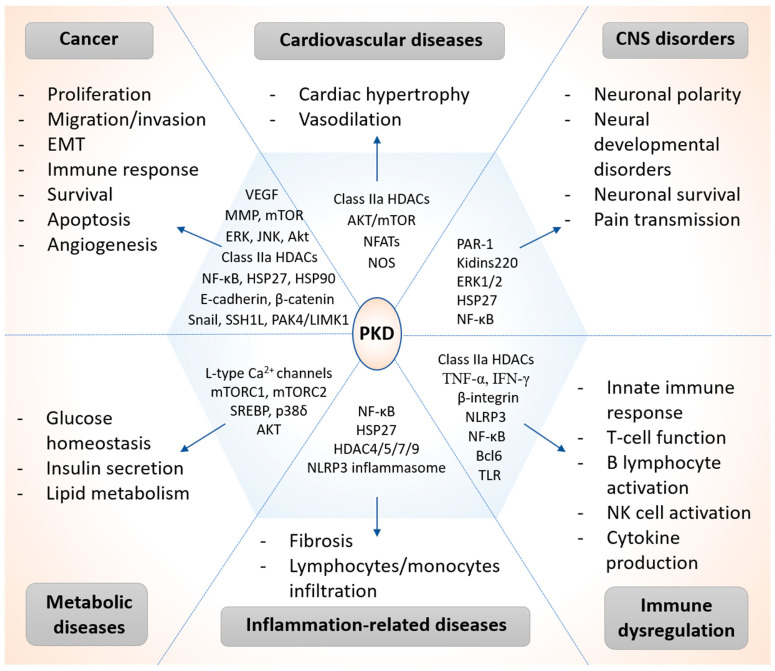
Versatile roles of PKD in human diseases. Schematic representation of key roles and major signaling targets of PKD in the pathogenesis of cancer and other human diseases. Abbreviations: central nervous system (CNS), epithelial-to-mesenchymal transition (EMT), natural killer (NK) cell, Toll-like receptor (TLR), nuclear factor of activated T cells (NFATs), sterol regulatory element binding protein (SREBP).

Emerging evidence indicates that PKD is involved in the regulation of a battery of cellular and pathophysiological conditions linked to a variety of human diseases, such as cancer [6,7], cardiovascular diseases [13,16], inflammatory diseases [39,40], and so on. Here, we have summarized recent studies on the signaling mechanisms and functional involvement of PKDs in major human health conditions, highlighting the key signaling proteins regulated by PKD in each pathological condition (Figure 3).

### 3.1. Cancer

PKD and its downstream targets are linked to the development and progression of human cancers, including those of the prostate, breast, pancreas, skin, stomach, etc. PKD has been extensively studied in multiple cancer relevant pathological processes. Our review will focus on the impact of PKD in pathways pertinent to several hallmarks of cancer, including enhanced growth, proliferative capacity [6,7,39,41,42], evasion of cell death [6,43,44,45], migration and invasion [41,42], angiogenesis [46,47], and immune modulation [6,48,49,50]. The role of each PKD isoforms in each of the pathological process will be reviewed and discussed (see a summary on the contributary roles of PKD isoforms in cancer in Table 1).

#### 3.1.1. Cell Growth and Proliferation

Uncontrolled proliferation is one of the most relevant hallmarks of cancer that leads to the formation of a tumor mass. PKD regulates cell proliferation in a variety of cellular systems. It was the earliest biological function characterized for PKD [95,96]. Pioneered by the Rozengurt group, it has been shown that a large number of mitogenic GPCR agonists, such as bombesin, vasopressin, endothelin, and bradykinin, can activate PKD1 through G_q_ and G_12_, leading to enhanced cell proliferation [95,97,98,99,100,101,102]. The downstream effectors include the class IIa histone deacetylases (HDACs) [103] and the β-catenin transcription factor [104]. Cell proliferation is controlled through regulation of the cell cycle. PKD has been identified as a novel cell cycle regulator at the G2–M and mitotic phase of the cell cycle in normal cells [105,106,107,108]. We have reported that PKD2 is activated during G2–M and that inactivation or depletion of PKD2 delays mitotic entry by inducing Aurora A kinase degradation [63]. Mechanistically, PKD2 colocalizes with Aurora A at the centrosome by binding to γ-tubulin, and knockdown of PKD2 causes defects in centrosome separation, an elongated G2 phase, mitotic catastrophe, and eventually cell death via apoptosis [63]. In line with our study, Kienzle et al. reported that knockdown of PKD1 and PKD2 leads to accumulation of cells in the G2 phase of the cell cycle and prevents cells from entering mitosis by blocking mitotic Golgi fragmentation via the Raf–MEK1 signaling pathway [106]. The Rey group reported that during mitosis, both PKD1 and PKD3 become phosphorylated in their activation loop at Ser^744^ and Ser^731^, respectively, in a PKC-dependent manner [105]. Further analysis revealed that all three activation loop-phosphorylated PKDs associated with the mitotic apparatus, including centrosomes, spindles, and midbodies [105]. PKD1 inhibition during mitosis results in formation of abnormal spindles, defects in chromosome alignment, and segregation, indicating that PKD1 activation is required for mitotic cell cycle transition [108]. The regulation of G2 and M implies that PKD may be a valuable target for inhibition of cancer cell proliferation. Indeed, depletion or inhibition of PKD has been shown to induce G1 or G2–M cell cycle arrest in a variety of cancer cells. For example, we have shown that depletion of PKD2 causes accumulation of cells in G2–M and delays the mitotic entry of prostate cancer cells [63]. We and others have also shown that the inhibition of PKD by multiple small-molecule inhibitors blocks prostate cancer cell proliferation by arresting cells in the G2–M phase of the cell cycle [55,56,64,109]. Similarly, abrogation of PKD2 induces G2–M arrest in lung adenocarcinoma cell lines [110]. Besides the regulation at G2–M, PKD2 abrogation could also induce G1 arrest of glioblastoma cells, correlating with the downregulation of cyclin D1 [111]. 

Beyond cell cycle regulation, MEK/extracellular-regulated protein kinase (ERK) is a major signaling hub through which PKD regulates tumor cell proliferation [53,54,57,58]. In particular, PKD1 has been shown to mediate neurotensin (NT)-induced cell proliferation by suppressing JNK/c-Jun activation and increasing the duration of NT-stimulated ERK activation in pancreatic cancer cells, resulting in the accumulation of c-FOS, DNA synthesis, and proliferation of pancreatic carcinoma cells [51]. PKD1 overexpression also promoted GPCR agonists bombesin and gastrin-releasing peptide-induced ERK1/2 activity and potentiated bombesin-stimulated tumor cell proliferation in head and neck squamous cell carcinoma [53]. Accordingly, pharmacological inhibition of PKD or PKD2 knockdown in colon cancer cell lines blocked Akt and ERK signaling and suppressed NF-κB activity, leading to G2–M arrest and induction of apoptosis in vitro along with reduced tumor growth in vivo [64]. PKD2, on the other hand, was shown to inhibit glioma cell proliferation by upregulating Golgi phosphoprotein 3 (GOLPH3) and inducing Akt activation [65]. PKD3 supports the proliferation of triple-negative breast cancer cell proliferation through mammalian target of rapamycin complex 1 (mTORC1)-S6 kinase 1 and the subsequent autophagic pathway [66]. Beyond the regulation of major signaling molecules, PKD also regulates transcription factors that are critical for tumor cell proliferation, such as c-MYC and class IIa HDACs [67,68]. Deletion of *PRKD3* by CRISPR/Cas9 in breast cancer cell lines suppressed phosphorylation of ERK1 and c-MYC and led to reduced cell proliferation in vitro and tumor growth in vivo [67]. Silencing PKD2 or PKD3 also significantly blocked HCC1806 triple-negative breast cancer cell proliferation, and in particular, PKD3 knockdown inhibited Hsp27 and HDAC4/5/7 phosphorylation [68]. Thus, PKD1–3 have all been reported to promote tumor cell growth and proliferation through both shared and distinctive signaling pathways. 

Although most studies support a pro-proliferative role of PKD in various biological systems, some have shown an opposite antiproliferative function of PKD1 in several cancers, including prostate [59,77], lung [62], and colon [61] cancer. PKD1 expression was shown to be downregulated in higher-grade colon cancer compared with non-neoplastic samples [61], and overexpression of PKD1 in human SW480 colorectal cancer cells inhibited cell proliferation, clonogenicity, and delayed tumor growth in a xenograft mouse model [61]. The antiproliferative effect was mediated through PKD1-induced nuclear exclusion of β-catenin and the subsequent decrease of its transcriptional activity [61]. Additionally, PKD1 was also found to be downregulated in non-small cell lung cancer patients with venous invasion or lymph node metastasis and loss of PKD1 enhanced the malignant potential of the tumor cells, possibly by negative regulation of mTORC1–S6K1 signaling [62]. Similarly, the Balaji group reported that PKD1 expression was reduced in metastatic prostate cancer samples but not in benign and primary tumor samples [59]. The authors also demonstrated that PKD1 interacts with β3 integrin to activate ERK signaling, which leads to increased secretion of matrix metalloproteinase (MMP)-2 and MMP-9, therefore suppressing prostate cancer cell proliferation as well as colony formation [59]. A later study by the same group showed that overexpressing PKD1 in prostate, breast, and colon cancer cell lines led to accumulation of cells in G1 phase, which may be mediated through the direct phosphorylation of all three cell-division cycle 25 proteins (Cdc25s) (A, B, and C) by PKD [60]. The antiproliferative effect of PKD could also be mediated through the binding of PKD1 with β-catenin and the subsequent inhibition of β-catenin-mediated proliferation [77]. Interestingly, PKD1 can also be repressed by oncogenes, providing a molecular basis for its downregulation. Specifically, it was found that nuclear β-catenin could recruit MYC and its obligate heterodimer MAX to form a transcription complex, which repressed *PRKD1* expression by binding to the promoter [112]. Taken together, there is compelling evidence that PKD1 could repress cell proliferation in certain tumor types, although the underlying molecular mechanisms are not fully understood.

Several genetically modified mouse models were developed to study PKD1 involvement in tumor initiation and progression in vivo. The Storz group generated a conditional PKD1 knockout mouse model to study the role of PKD in the initiation of pancreatic cancer [52]. In transgenic KC mice (p48^cre^; Kras^G12D^), expression of active Kras^G12D^ in pancreatic cells can drive the formation of acinar-to-ductal metaplasia (ADM) and further the progression to pancreatic intraepithelial neoplasia (PanIN). It was found that PKD1 expression was upregulated in regions of ADM and in PanINs in these transgenic mice [52]. By crossing PKD1 knockout (PKD^-/-^) mice with the KC mice, the authors generated acinar cell-specific PKD1-knockout KC mice (p48^cre^; Kras^G12D^; PKD1^-/-^), which showed that loss of PKD1 in pancreatic acinar cells significantly decreased active Kras^G12D^-induced formation of PanIN lesions [52]. These results revealed a functional role of PKD1 in driving ADM reprogramming and progression to PanINs. In another study by the Ghazizadeh group, mice with epidermis-targeted PKD1 deletion were generated and displayed a normal skin phenotype, indicating that PKD1 is dispensable in skin development and homeostasis [41]. However, when the dorsal skin was wounded, PKD1-knockout mice displayed delayed wound re-epithelialization, reduced proliferation, and migration of keratinocytes at the wound edge compared to the control mice [41], implying a potential critical pro-proliferative role for PKD1 in skin carcinoma. The Balagi group also generated a prostate-specific PKD1-knockout and a phosphatase and tensin homolog (PTEN)/PKD1 double-knockout mouse line with expression of different fluorescent proteins (Prorainbow mouse) for the purpose of cell lineage tracing in both normal and malignant prostate development [113]. The authors commented that the PKD1-knockout Prorainbow mice presented normal prostate size and pathological phenotype similar to those of control littermates [113], indicating that loss of PKD1 did not impact prostate development. However, the in vivo tumor-suppressive function of PKD1 in prostate cancer, particularly in the PTEN/PKD1 double-knockout mice, has not been reported.

The seemingly opposing effects of PKD1 on cell proliferation in different cancer cells are both interesting and intriguing, suggesting that other unknown factors or the cellular environment may alter the behaviors of PKD1 in cancer cells. Certainly, these discrepancies could also be explained by the use of different experimental systems, reliability of antibodies in detecting PKD expression and activity, and limited tissue samples examined, etc. Many of these studies rely on overexpression of PKD1 in cells, which could be problematic and requires further validation using other more physiological approaches. The lack of animal models to verify the function of PKDs in vivo is also a major drawback in the current state of PKD research in cancer.

#### 3.1.2. Cell Survival and Apoptosis

Besides uncontrolled tumor cell growth, evasion of programmed cell death (apoptosis) is another major driving force of tumor progression. All three PKDs have been reported to promote tumor cell survival. The major pathways through which PKD promotes tumor cell survival are NF-κB [8,72], ERK1/2 [64,73], Akt [64,73], and heat shock protein 90 (HSP90) [45] signaling. PKDs are known to protect cells from oxidative stress-induced cell death by activating the NF-κB transcription factor [34]. The production of reactive oxygen species at the mitochondria results in tyrosine phosphorylation of PKD at Tyr^463^ in the PH domain [34]. PKD then activates NF-κB and induces MnSOD to promote cell survival, as described above [34,36]. PKD2 protects tumor cells from apoptotic cell death by activating NF-κB signaling in several cancers [8,71,72]. For example, in human myeloid leukemia cells, PKD2 is constitutively tyrosine-phosphorylated by the oncogenic Bcr–Abl fusion protein, a driver of chronic myeloid leukemia and a mediator of Bcr–Abl-induced NF-κB activation [72]. In colon cancer cells, PKD inhibition in combination with regorafenib (an oral multikinase inhibitor with antiangiogenic activity) resulted in enhanced apoptosis through downregulating ERK, AKT, and NF-κB signaling activities [71]. Besides NF-κB signaling, PKD also regulates the expression of other prosurvival proteins. In pancreatic adenocarcinoma cells, PKD1 expression correlated with resistance to CD95-induced apoptosis and promoted cell survival through the induction of the antiapoptotic proteins survivin and c-FLIP_L_ [70]. In prostate cancer cells, PKD2 played an antiapoptotic role in phorbol ester-induced apoptosis in androgen-sensitive prostate cancer cells through the ERK1/2 and NF-κB pathways [8]. Furthermore, PKD2 interacted with and was stabilized by the HSP90 chaperone protein in several human cancer cell lines to support tumor survival [45]. Specifically, both short hairpin RNA (shRNA)-mediated abrogation of HSP90 isoforms (HSP90ɑ and HSP90β) and pharmacologic inhibition of HSP90 by PU-H71 and STA-9090 resulted in decreased PKD2 protein levels and enhanced apoptosis in lung, pancreatic, and breast cancer cell lines [45]. Conversely, ectopic expression of PKD2 protected cancer cells from HSP90 inhibition-induced apoptotic effects in two in vivo mouse models [45]. In addition, increased PKD3 expression and PKD3 nuclear accumulation were found in androgen-independent prostate cancer cell lines compared with androgen-dependent LNCaP cells [73]. Overexpression of PKD3 in LNCaP cells blocked phorbol ester-induced apoptosis and also inhibited phorbol ester-induced downregulation of Akt activity, demonstrating the prosurvival effects of PKD3 [73]. Taken together, members of the PKD family in general promote tumor cell survival by activating major survival pathways, such as the NF-κB signaling pathway, to evade apoptosis induced by cellular stresses.

Cytotoxic chemotherapy drugs induce apoptotic cell death in cancer cells to prevent tumor progression. One of the major obstacles for chemotherapeutic treatment in human cancers is the development of drug resistance. PKDs have been implicated in the development of drug resistance in multiple cancers. PKD1 overexpression enhanced cell viability and resistance to gemcitabine through activating glucose transporter 1 and mTORC1 to increase metabolic adaptation in pancreatic cancer cells [69]. Furthermore, PKD2 was identified as an important regulator of drug resistance and P-glycoprotein expression in paclitaxel-treated breast cancer cells [114]. Knockdown of PKD2 in these cells resulted in a significant decrease in resistance to paclitaxel treatment [114]. In addition, PKD3 was identified as a valid target to overcome drug resistance against RAF and MEK inhibitors in melanoma cell lines [115]. Although this area of research remains sparse, current findings support targeted inhibition of PKD as a potential strategy to overcome therapeutic resistance in cancer.

#### 3.1.3. Cell Adhesion, EMT, Migration, and Invasion

The epithelial-to-mesenchymal transition (EMT) is a biological process by which epithelial type cells lose their polarization and cell–cell adhesion through cytoskeleton reorganization and undergo distinct biochemical changes to become mesenchymal-type cells with enhanced migratory and invasive properties [116]. A hallmark for EMT is the loss of E-cadherin, a transmembrane protein that mediates cell–cell adhesion in epithelial cells. Negative regulation of *CDH1*, the gene that encodes E-cadherin, by transcription factors Snail, Slug, ZEB1, and ZEB2 resulted in the loss of E-cadherin [117]. E-cadherin exerted its antiproliferative, anti-invasive, and antimetastatic properties through binding to β-catenin to form a protein complex and interacting with the actin and microtubule cytoskeleton [117,118]. Multiple lines of evidence have indicated that PKD could regulate EMT through modulating the E-cadherin/β-catenin complex and EMT regulatory transcription factors in epithelial cells. The Storz group demonstrated that PKD1 was expressed constitutively in murine mammary gland epithelial cells and prevented transition to a mesenchymal phenotype [119]. The authors show that PKD1-mediated phosphorylation of Snail, a transcriptional repressor of E-cadherin, at Ser^11^ reduced the ability of Snail to bind to its corepressor protein Ajuba, and therefore inhibited the ability of Snail to repress E-cadherin expression and consequently blocked EMT [119].

Cells that have undergone EMT due to loss of E-cadherin display enhanced motility, a process that requires concerted regulation of multiple cellular events including actin cytoskeletal remodeling. During migration, cells move with extending protrusions at the leading edge and a retracting tail at the rear; both are associated with actin remodeling events which are regulated by a group of actin-related proteins such as cofilin, vasodilator-stimulated phosphoprotein (VASP), profilin, and actin-related protein-2/3 complex (ARP2/3) [120,121]. Cell migration is achieved when the actin-binding protein cofilin slices actin filaments at the leading edge of motile cells, generating a supply of actin monomers, and orchestrating the formation of WAVE-2–cortactin–ARP2/3 complex, which ultimately creates an expanded, branched network of F-actin [120]. Cofilin can be inactivated through phosphorylation at Ser^3^ by kinases such as LIM domain kinase 1 (LIMK1) (because it can no longer bind to actin), and cellular migration is suppressed when cofilin is phosphorylated, whereas cell motility is restored through cofilin dephosphorylation by phosphatases such as slingshot protein phosphatase 1L (SSH1L) [122,123]. Studies by the Storz group show that overexpression of constitutively active PKD1 significantly decreased, whereas overexpression of inactive mutants of PKD1 increased cell migration [124]. In another study, they further demonstrated that PKD1 phosphorylated SSH1L at Ser^978^ in its actin-binding motif, which then generated a 14-3-3 binding motif that blocked localization of SSH1L to F-actin in the lamellipodium, blocking cofilin dephosphorylation and thereby inhibiting cell migration [124]. PKD1 also regulated cofilin activity by phosphorylating/activating p21-activated kinase 4 (PAK4), an upstream protein kinase for LIMK1 [125]. Activation of PAK4/LIMK1 signaling led to the accumulation of phosphorylated inactive cofilin in cells and consequently blocked cell migration [125]. Besides the regulation of cofilin, PKD1 also blocked cell migration through phosphorylating the Ras effector protein Rab interactor 1 (RIN1) at Ser^292^, a phosphorylation site that controls the RIN1-mediated inhibition of cell migration by modulating Abl kinases’ activation [126]. Moreover, PKD1 inhibited cell motility through regulating focal adhesion dynamics and filopodium formation, which are mediated through the phosphorylation of phosphatidylinositol-4-phosphate 5-kinase type-1γ (PIP5K1γ) [127] and VASP, respectively [128].

During the tumorigenic process, PKD1 is thought to function by maintaining the epithelial phenotype, while malignant cells gain motility by downregulating PKD1 expression [14,42,82,129]. PKD1 negatively regulates cancer cell motility through multiple mechanisms including inactivating transcription factor Snail [74,75], phosphorylating adherens junctional proteins (E-cadherin and β-catenin) [76,77] and metastasis-associated protein 1 (MTA1) [78], regulating integrin trafficking [79], stabilizing F-actin filaments at leading edges and filopodia [81,88,124,125,126,128], and repressing the expression of MMPs [80]. In prostate and breast cancer cells, PKD1 phosphorylated Snail, resulting in the nuclear export and proteasomal degradation of Snail and subsequent inhibition of EMT [74,75]. In addition to transcriptional regulation of E-cadherin, PKD1 also phosphorylated E-cadherin directly. Jaggi et al. showed that PKD1 interacted with E-cadherin at cell junctions in prostate cancer cells [76]. More interestingly, phosphorylation of E-cadherin by PKD1 was associated with increased cellular aggregation and decreased cellular motility in prostate cancer [76]. The same group also demonstrated that PKD1 interacted with and phosphorylated β-catenin and therefore controlled the formation of E-cadherin-mediated cell adhesion in epithelial cells [77]. In a recent study, Ganju et al. identified another PKD1-interacting substrate, metastasis-associated protein 1 (MTA1), in prostate cancer [78]. The authors showed that PKD1 phosphorylated MTA1 and triggered polyubiquitination and proteasomal degradation of MTA1, therefore inhibiting tumor cell migration and invasion [78]. The authors further demonstrated a reverse correlation between PKD1 and MTA1 expression in a transgenic adenocarcinoma of the mouse prostate (TRAMP) model and in samples of human prostate cancer, in which PKD1 expression decreased, whereas MTA1 expression increased with progressed tumor stage [78]. Additionally, PKD1 promoted αvβ3 integrin recycling through phosphorylating rabaptin-5 and inhibited both breast cancer and colorectal cancer cell invasion into fibronectin-rich matrices [79]. In breast cancer cell lines, overexpression of constitutively active PKD1 repressed tumor cell migration, whereas downregulating PKD1 led to increased cell migration [81,124]. For example, Philipp et al. showed that the loss of PKD1 decreased cofilin phosphorylation and induced chemotactic migration of breast cancer cells in an SSHL1-dependent manner [81]. PKD1 also regulated the expression of MMPs, which are a class of proteases that mediate cell migration through extracellular matrix (ECM) degradation. It was reported that PKD1 inhibited breast cancer cell invasion by negatively regulating the transcription of several MMPs, including MMP-2, MMP-7, MMP-9, MMP-10, MMP-11, MMP-13, MMP-14, and MMP-15 [80]. Taken together, PKD1 is a negative regulator of EMT, cell migration, and invasion.

In contrast to PKD1, PKD2 and PKD3 appear to act in the opposite manner and positively regulate EMT and migration/invasion through multiple mechanisms involving the regulation of cofilin [88], NF-κB and urokinase-type plasminogen activator (tPA) expression/activation [83], MMP and integrin expression [84,85,130,131], and the PI3K/AKT/glycogen synthase kinase 3 beta (GSK-3β)/β-catenin pathway [86]. In fact, it has become increasingly clear that PKD2 and PKD3, opposite to PKD1, associate with more malignant cancer phenotypes and promote tumorigenesis and progression. Our earlier work has demonstrated increased expression levels of PKD3 in human prostate cancer samples when compared to normal prostate tissues, and there was a correlation between PKD3 nuclear localization and higher tumor grade [73]. Zou et al. showed that endogenous PKD2 interacted with and phosphorylated IKKβ in invasive prostate cancer cells and was responsible for the nuclear translocation and activation of the p65 subunit of NF-κB through phosphorylation of S276 on p65, whereas PKD3 was responsible for S536 phosphorylation on p65 and deactivation of HDAC1 [83]. These PKD2- and PKD3-mediated signaling events coordinated to promote prostate cancer cell invasion [83]. Our group investigated functional roles of PKD3 in prostate cancer cell motility using both a stable inducible knockdown cell model and a transient knockdown system via multiple small interfering RNAs (siRNAs) [87]. We found that the silencing of endogenous PKD3 significantly reduced prostate cancer cell migration and invasion [87]. Mechanistic studies indicated that depletion of PKD3 blocked the secretion of MMP-9 and several tumor-promoting cytokines, such as IL-6, IL-8, and GROα, without altering their mRNA levels [87] and that inducible depletion of PKD3 in a subcutaneous xenograft model decreased levels of intratumoral GROα in mice [87]. Similarly, PKD2 and PKD3, but not PKD1, were expressed in the highly metastatic breast cancer cell lines MDA-MB-231 and MDA-MB-468 [88,130,132]. PKD3 was constitutively active in MDA-MB-468 breast cancer cells under normal growth conditions, and this basal activity was sufficient to stimulate PAK4/LIMK signaling without affecting SSH1L activity, which contributed to a functional cofilin activity cycle and directed cell migration by activating PAK4 [88]. In addition to regulating the cofilin cycle, PKD3 can also stimulate migration through phosphorylating G-protein-coupled receptor kinase-interacting protein 1 (GIT1), whose dynamic localization to sites of cytoskeletal remodeling was critical in the regulation of cell spreading and migration [89]. In line with the studies on PKD3, Yasuhito et al. showed that siRNA-mediated knockdown of PKD2 efficiently inhibited Matrigel invasion of MDA-MB-231 cells [130]. A similar effect was observed in another breast cancer cell line, in that siRNA-mediated knockdown of PKD2 inhibited migration of MCF7 cells [133]. Meanwhile, PKD2 knockdown in lung adenocarcinoma cell lines significantly reduced the expression of mesenchymal markers (N-cadherin, vimentin) and the transcription factors that stimulated EMT (Twist, Snail), along with inhibiting cell migration, invasion, and proliferation, indicating that PKD2 promotes the EMT and metastatic potential of lung adenocarcinoma [110]. In another study by the Seufferlein group, PKD2 was upregulated in pancreatic cancer, and ectopic expression of PKD2 significantly enhanced invasion of pancreatic cancer cells in the surrounding three-dimensional ECM through stimulating expression and secretion of MMP-7 and MMP-9 [84]. PKD2 has also been reported to promote migration and invasion by regulating MMP-1 and integrin expression in glioblastoma [85]. In addition, PKD2 is upregulated in hepatocellular carcinoma (HCC) and is correlated with the metastasis of HCC [86]. The authors demonstrated that PKD2 positively regulated TNFα-induced EMT and invasion of HCC through promoting the PI3K/Akt/GSK-3β signaling cascade [86]. Overall, in contrast to the negative regulation of EMT, migration, and invasion by PKD1, PKD2 and PKD3 generally promote tumor metastasis by stimulating EMT and tumor cell migration/invasion in many cancer types.

#### 3.1.4. Angiogenesis

Angiogenesis, the growth of new capillary blood vessels from the pre-existing vasculature, plays pivotal physiological roles in embryonic development, wound healing, tissue regeneration, and placental development [134,135,136]. However, when dysregulated, it can also contribute to oncogenic, ischemic, infectious, and inflammatory diseases [134,137]. Tumor development requires angiogenesis, which brings oxygen and nutrient supplies to and metabolic wastes away from tumor cells.

The angiogenic response is strictly controlled by pro- and antiangiogenic molecules including growth factors, MMPs, cytokines, and integrins [138,139]. Specifically, the major signaling molecule that regulates angiogenesis is vascular endothelial growth factor (VEGF), which is commonly found to be induced by hypoxia or oncogene signaling. VEGF then signals through its cognate receptor VEGF receptor-2 (VEGFR-2) and induces downstream signals that stimulate endothelial cell survival, proliferation, migration, and differentiation [140]. A recent study demonstrated that silencing PKD1 or PKD2 in endothelial cells enhanced association of transcription factor AP2β with the VEGFR-2 promoter, decreasing VEGFR-2 transcription [141]. Emerging studies have shown that PKDs regulate both hypoxia-induced VEGF secretion by tumor cells and VEGF-stimulated tumor angiogenesis [46,90,92,141,142]. Overexpressing PKD1 in pancreatic cancer cells significantly increased their secretion of the proangiogenic factors VEGF and CXC chemokines, and thereby promoted anchorage-independent growth, invasion, and angiogenesis in human pancreatic cancer [90]. The Ren group reported that diet-induced obesity could promote breast tumor progression via lysophosphatidic acid/PKD-1 signaling-mediated angiogenesis [91]. PKD2 was a crucial mediator of hypoxia-induced VEGF-A expression and secretion in pancreatic tumor cells and promoted tumor-driven blood vessel formation and tumor growth in vitro and in vivo [92]. Because HSP90 bound and stabilized PKD2 in cancer cells, inhibition of HSP90 led to degradation of PKD2, thereby indirectly impacting the proangiogenic function of PKD2 under hypoxic conditions by blocking HIF1α accumulation and NF-κB/VEGF-A signaling activity [44,45]. Additionally, both PKD2 and PKD3 have been shown to contribute to mast cell recruitment and tumor angiogenesis in the prostate cancer microenvironment [47]. However, in this study, the authors commented that depletion of PKD2 or -3 had no effect on VEGF secretion from prostate cancer cells or mast cells; instead, a role of PKD2 and PKD3 on the secretion of stem cell factor and chemokine ligand 5 and C-C motif chemokine 11 expression was implicated in the response [47]. Overall, all three PKDs have been shown to promote angiogenesis through multiple mechanisms with VEGF as a major signaling hub. Therefore, inhibition of PKD may serve as a potential effective way to interrupt tumor angiogenesis and block tumor progression.

#### 3.1.5. Immune Responses in Cancer

PKD2 is the major PKD isoform expressed in human and mouse T and B lymphocytes, thymocytes, and spleen cells [143]. Type 1 interferons (IFN-α/β) play a critical role in modulating the immune responses against infectious agents and tumors [94]. IFNα activated PKD2 through tyrosine phosphorylation at Tyr^438^; this molecular event was also necessary for efficient serine phosphorylation and degradation of IFNAR1 and consequently restricted magnitude and duration of cellular responses to IFN-α/β [94]. Interestingly, PKD2 was identified as an important regulator of programmed death ligand-1 (PD-L1) surface expression downstream of IFN-γ in human oral squamous carcinoma cells [93]. Inhibition of PKD2 activation not only decreased PD-L1 expression, but also reduced drug resistance in chemotherapy [93]. In a recent study, PKD3 was also found to regulate the expression of PD-L1 induced by IFN-γ in oral squamous cell carcinoma [50]. These studies imply that PKD may modulate the immune response of tumor cells, representing a novel potential target in cancer immunotherapy.

### 3.2. Cardiovascular Diseases

PKD is an important modulator of stress signaling in the heart and regulates many biological processes in cardiac myocytes, such as gene expression, cell survival, excitation-contraction coupling, and metabolism [13]. PKD1 is activated in the heart in response to hypertension, pressure overload, and chronic neurohormonal stimulation [144,145,146,147]. The Olson group reported that cardiac-specific deletion of PKD1 diminished cardiac hypertrophy and improved cardiac function in response to pressure overload, chronic adrenergic stimuli, or angiotensin II signaling in mice [146]. Mechanistically, PKD1 phosphorylated class IIa histone deacetylases (HDAC4, -5, -7, -9) (known negative regulators of pathological cardiac remodeling through binding and transcriptional repression of the myocyte enhancer factor-2 (MEF2) transcription factor) and triggered their nuclear export, resulting in MEF2 derepression and cardiac remodeling [146]. In a separate study using a transverse aortic constriction-induced cardiac hypertrophy mouse model, the authors found that PKD1 contributed to cardiac hypertrophy through inhibiting AKT/mTOR regulated cardiac autophagy [144]. In addition, PKD1 can also phosphorylate endothelial nitric oxide synthase and regulate vascular endothelial growth factor-mediated angiogenesis [13]. For example, Aicart-Ramos et al. showed that PKD specifically phosphorylates recombinant endothelial nitric oxide synthase on Ser^1179^, leading to its activation and a concomitant increase in nitic oxide synthesis [148]. Moreover, inhibition of PKD in mice results in an almost complete disappearance of the vascular endothelial growth factor-induced vasodilation of the carotid artery [148]. Although PKD1 is by far the most abundant isoform in the heart, PKD3 has also been reported to mediate glucose uptake and account for the morphological and functional changes as seen during the development of cardiac hypertrophy driven by cardiac transcription factors [149,150]. Specifically, Li et al. demonstrated that PKD3 is required for the upregulation of the cardiac transcription factors including nuclear factor of activated T cells (NFATs), NK family of transcription factor 2.5 (Nkx2.5), and GATA4, which drive cardiac hypertrophy [149]. Taken together, the abnormally activated PKD, by altering the transcriptomic landscape of the cardiomyocytes, drives cardiac remodeling and represents an attractive potential therapeutic target to treat cardiovascular diseases such as stress-induced cardiac hypertrophy and heart failure.

### 3.3. CNS Disorders

Establishing and maintaining neuronal polarity is of great importance for neuronal signaling and function, and neuronal polarization is dependent on the reorganization of trafficking from the trans-Golgi network to plasma membrane [151,152]. Both PKD1 and PKD2 are known to specifically regulate basolateral membrane trafficking in polarized epithelial cells and are important players in the generation of epithelial polarity. Yin et al. demonstrated that PKD1 and PKD2, but not PKD3, modulated the polarized mode of hippocampal neurons [153]. The authors reported that PKD1 and PKD2 siRNAs disrupted polarized membrane trafficking and markedly increased the percentage of neurons with multiple axons compared to transfections with control siRNAs. On the contrary, transfection with dominant-negative PKD3 had no effect on neuronal polarization [153]. Later studies further confirmed that PKD1 regulated neuronal polarity through its activity in the Golgi apparatus, which allows PKD1 to regulate the sorting, packaging, and targeting of different proteins and suppress the endocytosis of dendritic membrane proteins [152,153]. PKD1 exerted these functions through phosphorylation of downstream effector proteins, including the scaffold protein Kidins220 and polarity protein PAR-1 [154,155].

PKDs have been implicated in several neural developmental disorders. PKD2 was reported in the pathogenesis of autism spectrum disorder (ASD) [156]. Matsumura et al. found that PKD2 was expressed at a very high level in neural stem cells in the embryonic cerebral cortex, and an ASD-associated de novo *PRKD2* mutation decreased the autophosphorylation of PKD2, and thereby PKD2 activity and the activity of its downstream effector kinase ERK1/2 [156]. The authors then concluded that ASD-associated *PRKD2* mutations can be a risk factor for ASD due to disrupting PKD2 cellular function [156]. Moreover, *PRKD2* deletion was found in Rett syndrome (RTT), another neurodevelopmental disorder affecting the nervous, musculoskeletal, and gastroenteric systems [157,158,159]. Whole exome sequencing and copy number variation analysis from a female child patient revealed a ~5 MB microdeletion at the long arm of the chromosome 14q12 region, which resulted in the deletion of a single copy of brain-specific genes including *PRKD1*, *FOXG1*, and *NOVA1* [158]. However, it remains to be determined if the deletion of *PRKD1* was causal to the disease phenotypes. Collectively, these studies imply that PKDs are important players in neurodevelopment and that the disruption of PKD expression or function may lead to neurodevelopmental diseases.

PKD also exerts neuroprotective functions under different stress conditions such as oxidative stress and cerebral ischemia, and both are major pathological causes for neurodegenerative diseases (Alzheimer’s, Parkinson’s, and Huntington’s diseases) and stroke [160]. Pose-Utrilla et al. reported that H_2_O_2_ treatment induced a short activation followed by a sharp inactivation of PKD that was triggered by calcium influx in cultured cortical neurons, resulting in the loss of NF-κB signals and impeding neuronal survival [161]. PKD is activated in ischemic stroke model [162]. Interestingly, heat-shock protein (HSP27), an established target that is known to exert cytoprotection against cerebral ischemia-induced apoptotic neuronal death [163], directly binds and is phosphorylated by PKD in neurons in response to ischemic/reperfusion injury [162]. Inhibition of PKD abolished HSP27-mediated neuroprotection against neuronal ischemic insult in a mouse model of transient cerebral ischemia—tMCAO [162]. Collectively, these studies imply that stress-induced activation of PKD is likely neuroprotective in pathological cerebrovascular diseases.

### 3.4. Metabolic Diseases

PKDs have been reported to play pivotal roles in metabolic regulation in various tissues and organs such as the liver, adipose tissue, skeletal muscle, and pancreatic β cells. PKD isoforms are well studied in the regulation of glucose homeostasis, insulin secretion, and lipid metabolism underlying metabolic disorders such as type 2 diabetes and obesity (summarized in other reviews [16,164]). The landmark discovery by the Ricci group showed that mice lacking the mitogen-activated protein kinase p38δ displayed improved glucose tolerance due to enhanced insulin secretion from pancreatic β cells and that deletion of p38δ resulted in dramatic activation of PKD and protected against hyperlipidemia-induced insulin resistance and oxidative stress-imposed pancreatic β cell apoptosis [24]. Mechanistically, p38δ exerts an inhibitory phosphorylation on PKD1, which regulated both stimulated insulin secretion and pancreatic β cell survival [24]. This study identified PKD1 as a crucial regulator of stimulated insulin exocytosis at the TGN, demonstrating for the first time the biological significance of PKD in protein trafficking at the TGN. However, several recent studies have revealed an opposite function of PKD1 and -2 in insulin secretion in pancreatic β cells. Bergeron et al. showed that β cell-specific knockout of PKD1 not only did not downregulate insulin secretion, but rather caused upregulation of insulin secretion and exacerbated hyperglycemia, hyperinsulinemia, and glucose intolerance in the PKD1-knockout mice fed with a high-fat diet [165]. Similarly, Xiao et al. showed that PKD2 deletion also triggered hyperinsulinemia, which preceded insulin resistance and metabolic disorders in the PKD2-deficient mice [166]. At the molecular level, PKD2 deletion promoted insulin secretion by β cells through increasing the expression and activity of L-type Ca^2+^ channels and subsequently augmenting high glucose- and membrane depolarization-induced Ca^2+^ influx, implying that downregulation of PKD2 may contribute to hyperinsulinemia and systemic insulin resistance that underlie metabolic disorders [166]. PKD3 may play a minor role in regulating insulin secretion from pancreatic β cells, since it has been shown that PKD1 and PKD2 are the predominant isoforms in the islets, while PKD3 is more abundant in the exocrine cells [167]. Nonetheless, PKD3 is an important regulator of glucose and lipid metabolism in other organs. A recent study by the Sumara group demonstrated that in the liver, PKD3 was the predominant PKD isoform expressed in hepatocytes and was activated by lipid overload [168]. Functionally, PKD3 suppressed insulin signaling, as indicated by the reduced Akt and mammalian target of rapamycin complex 1 and 2 (mTORC1 and mTORC2) activities, and promoted insulin resistance. PKD3-knockout mice also showed improved hepatic insulin-induced glucose tolerance. Additionally, hepatic deletion of PKD3 increased triglyceride and cholesterol content in the livers via sterol regulatory element binding protein (SREBP), providing a negative feedback regulation on hepatic lipid production [168]. There is growing interest in the role of PKD in metabolism. Current studies have established the crucial role of PKD in regulation of insulin secretion, insulin signaling, and lipid synthesis. However, the exact function of each isoform and signaling mechanisms in glucose homeostasis and energy metabolism remain to be fully defined. Although there are currently no ongoing clinical trials concerning the PKD family in metabolic disorders, targeting PKD isoforms may still represent a promising strategy for the treatment of obesity and diabetes.

### 3.5. Inflammation-Related Diseases

PKD is a major player in inflammatory processes that are coupled with the development of many diseases. PKD is required for the production of proinflammatory cytokines and chemokines in tumor cells [87], immune cells [169,170,171], myofibroblasts [172], epithelial cells [173], and endothelial cells [174] in response to various stimuli. PKD regulates the migration and infiltration of lymphocytes [175] and monocytes [176] and modulates activities of NF-κB [72], HSP27 [177], and COX-2 [172]—all key regulators of inflammatory responses. Recently, PKD has also been shown to phosphorylate the Nod-like receptor protein 3 (NLRP3) inflammasome, a critical mediator of innate immunity and inflammatory responses, and release it from mitochondria-associated endoplasmic reticulum membranes adjacent to the Golgi membranes, resulting in inflammasome activation, which is crucial for maintaining efficient host defense in complex organisms [178].

In line with the important role of PKD in inflammation, PKD has been implicated in the following inflammatory diseases: pancreatitis [179,180], inflammatory bowel disease [177], inflammation-induced hyperalgesia [181], hypersensitivity pneumonitis [169], allergic inflammatory diseases [171], bacterial infection [182], viral infection-induced airway inflammation [183], and Sjogren’s syndrome-related inflammation [175]. Specifically, the Yi group reported that *Saccharopolyspora rectivirgula* induced activation of PKD1 in innate immune cells in the lung and that PKD1 was indispensable for *Saccharopolyspora rectivirgula*-mediated activation of mitogen-activated protein kinases and NF-κB, as well as expression of various proinflammatory cytokines and chemokines [169]. Pharmacological inhibition of PKD attenuated early events of experimental pancreatitis in isolated rat acini and blocked NF-κB activation and attenuated pancreatic injury in animal models of pancreatitis [179]. In addition, a study using human colonic myofibroblasts (an activated or differentiated form of fibroblasts that responds to inflammatory signals and contributes to inflammation-associated fibrosis) showed that bradykinin stimulated migration of these cells via PKD-mediated activation of COX-2 and HSP27, implying a role of PKD in inflammatory bowel disease [177]. PKD isoforms are also reported to mediate protease-induced neurogenic inflammation and pain [184]. Taken together, PKD may be a promising target for anti-inflammatory therapies.

Fibrosis describes the pathological process of tissue thickening or scarring accompanied by the deposition of collagen and other ECM components [185]. In contrast to acute inflammatory response, fibrosis is the consequence of chronic inflammation in response to various pathologic conditions such as viral/bacterial infection, allergic response, tissue repair, radiation, toxins, and autoimmune reactions [185]. PKD has been implicated in the progression of heart, liver, and lung fibrosis [146,186,187,188,189]. The Olson group reported that cardiac-specific deletion of PKD1 in mice resulted in diminished fibrosis and improved cardiac function in response to pressure overload through regulating HDACs and MEF2 [146]. Sin et al. demonstrated that impeding the nuclear translocation of PKD1 was protective against accumulation of fibrillar collagen and development of the cardiac fibrosis that accompanied cardiac remodeling following pressure overload [28]. In addition to its role in the heart, Gan et al. found that PKD was increased and activated in lung epithelial cells and macrophages in idiopathic pulmonary fibrosis and may participate in the pathogenesis of this disease [186]. It has also been demonstrated that global *Prkd3*-knockout and myeloid cell-specific *Prkd3*-knockout mice developed spontaneous liver fibrosis [187]. It was found that PKD3 deletion drives liver fibrosis through activating profibrotic macrophages [187]. Overall, it is evident that PKDs play an indispensable role in fibrotic disorders. Isoforms of PKD are differentially implicated in fibrosis of different organs, with PKD1 and -2 promoting and PKD3 inhibiting fibrosis. It remains to be determined if these effects are organ- or isoform-specific. More in-depth investigation on the mechanisms of specific PKD isoforms in fibrotic processes will help unveil their importance as potential novel therapeutic targets for this disease.

### 3.6. Immune Dysregulation

Since the discovery of PKD as a DAG receptor and a key regulator of NF-κB, a transcriptional factor involved in both innate and adaptive immune response, its involvement in immune response has been intensely investigated [190,191]. PKDs are expressed in B and T lymphocytes, macrophages, mast cells, and natural killer cells [17,191]. PKD has been shown to be involved in the regulation of innate immune response through the MyD88-dependent Toll-like receptor (TLR) signaling pathway [17,192] and by regulating NLRP3 inflammasomes at the Golgi [178], class II HDACs in lymphocytes [193,194], and β1 integrin activity in T lymphocytes [195]. PKD2 is also an important signaling component in natural killer cell activation, which establishes the first line of innate immune defense against pathogen-infected cells [48]. PKD2 also plays a unique role in controlling T-cell functions during adaptive immune responses, since PKD2 catalytic activity is required for effective cytokine production after T-cell receptor activation [143]. PKD is an important mediator of B-lymphocyte activation [196]. Sokol et al. reported that activation of PKD was necessary for the synergy between the B-cell Ag receptor and tumor necrosis factor receptor CD40 in B lymphocytes, and the synergetic interaction provided critical signals for B-cell differentiation, isotype switching, and B-cell memory [196]. Evidence suggested that PKD was also involved in T-cell development and function [197,198]. Ishikawa et al. demonstrated that PKD was required for thymic selection during T-cell development [199]. The authors generated T-cell-specific PKD-deficient (PKD2/PKD3 double-deficient) mice and found that the generation of CD4+CD8- and CD4-CD8+ single-positive thymocytes was impaired in these mice [199].

A recent study by the Beutler group reported an excessive T follicular helper cell (TFH) development, germinal center formation, germinal center B-cell activation, and anti-DNA antibodies’ production with age in *Prkd2*-/- mice [200]. The authors demonstrated that PKD2 bound and phosphorylated Bcl6 (a transcription repressor) to limit Bcl6 nuclear translocation in CD4+ T cells, resulting in excessive cell autonomous TFH development [200]. TFH are known to provide signals to B cells to initiate the humoral immune response to most protein antigens, such as germinal center formation, somatic hypermutation, and affinity maturation [201]. Thus, increased TFH can increase autoantibody or IgE production, leading to autoimmune or allergic diseases [202,203,204]. Therefore, more in-depth investigation on the role of PKD in T-cell responses will help to unveil its importance as a novel therapeutic target.

## 4. Targeted Inhibition of PKD in Diseases

With growing evidence supporting an important role of PKD in cancer and other diseases, the development of targeted therapies against aberrant PKD activities has gained considerable interest. Selective, potent, and structurally distinct pan-PKD small-molecule inhibitors have been reported, including CID755673 and analogs from our group [205,206,207,208,209], 2,6-naphthyridine and bipyridyl inhibitors and their analogs [210,211,212], as well as 3,5-diarylazoles from Novartis [213] and CRT0066101 [214] and CRT5 from Cancer Research Technology Ltd. [215], which all showed nanomolar inhibitory activities towards PKD. Among them, CRT0066101 is the most potent and efficacious PKD inhibitor by far, with demonstrated in vivo antitumor activity in pancreas, colon, breast, and bladder cancer models [64,109,214,216,217]. Additionally, CRT0066101 and CID755673 also showed efficacy in experimental models of acute pancreatitis [179,180], and CID755673 in diabetes and diabetic cardiomyopathy models [218,219]. Although currently there are no clinical trials on PKD inhibitors, these encouraging in vivo results suggest that targeted inhibition of PKD may be a viable therapeutic strategy for diseases with deregulated PKD signaling.

## 5. Perspectives and Future Directions

Through persistent efforts of many research groups, our understanding of PKD has advanced enormously over the past two decades. It has become increasingly clear that PKD is at the forefront of the DAG signaling network and plays pivotal roles in regulating many essential cell functions, such as cell proliferation, movement, secretion, contractility, and metabolism. Many of these functions stem from the crucial role of PKD in regulating vesicle trafficking at the TGN, actin dynamics, and gene transcription. In many of the biological systems examined, PKD appears to function as a key sensor for a variety of cellular stresses and nutrients, and its activation by these cues triggers compensatory adaptive responses that serve to maintain or strengthen normal cell functions. However, chronically, these adaptive responses often result in various pathological conditions and for PKD, the outcomes, for example, could include stress-induced cardiac hypertrophy [16] or high-fat-diet-induced hyperinsulinemia and glucose intolerance [165,220].

Among all the diseases discussed in this review, the role of PKD in cancer is by far the most intensively studied, and yet still the least understood. Aberrant PKD expression and activity have long been demonstrated in a variety of cancers, and increasing evidence supports the involvement of PKD in almost all aspects of tumor development, growth, evasion of apoptosis, angiogenesis, invasion and metastasis, and chemoresistance. Recent new studies have also linked PKD to immune modulation [50], microenvironment changes [47,91], and metabolic rewiring [221]. These multifaceted roles of PKD in cancer make it an appealing therapeutic target for cancer treatment. However, the therapeutic benefit of targeting PKD will likely be complicated by the differential roles of PKD isoforms in cancer; in particular, PKD1 has been shown to inhibit tumor cell proliferation in certain cancers and exert negative regulation on EMT, cell migration, and invasion. It is unclear if these differences at the cellular level can be extended to in vivo settings, and the development of tissue-specific PKD mouse models of cancer will be instrumental to address these questions. Meanwhile, despite the high sequence homology, there are unique structural features in each PKD isoform, which may account for the isoform-selective effects of PKD in specific cellular environments. The exact mechanisms underlying their distinct functionalities remain to be determined.

Besides cancer, PKD has also been studied extensively in the heart and the immune system, followed by the vasculature, CNS, and muscle. Other emerging areas include metabolic regulation in the liver, adipose tissue, skeletal muscle, and pancreas. Studies in these areas clearly demonstrate an important role of PKD in the pathogenesis of several major diseases, for example, stress-induced cardiac remodeling, obesity, diabetes, ischemic stroke, neurodevelopmental disorders, various infectious diseases, fibrosis, and autoimmune disorders (see Table 2 for a summary on the roles of PKD isoforms in other human diseases). It is important to note that a repeated theme in the pathogenesis of these diseases is the activation of PKD by various stresses and the downstream modulation of multiple common signaling nodes, including NF-κB, VEGF, ERK, Akt/mTOR, class IIa HDACs, integrin, and PI4KIIIβ. More studies are needed to examine the in vivo functions of PKD in different disease models and evaluate the therapeutic value of targeting PKD in these pathological conditions. Moreover, in light of the distinct roles of PKD isoforms in various pathological processes, it has become increasingly desirable to develop PKD isoform-selective small molecule inhibitors or even activators. Besides small molecules, other strategies, such as peptide- or nucleotide-based drugs, could also be exploited for more selective targeting of PKD. Additionally, identifying effective drug combinations of PKD inhibitors will not only reduce their toxicity and enhance their therapeutic efficacy, but also broaden the potential clinical usage of these inhibitors.

In summary, although significant progress has been made in our understanding of PKD, much more work is needed in order to clarify the different roles of PKD in various biological processes and the underlying mechanisms. The use of large-scale genome sequencing technologies, interrogation of various omics data, and analysis of large cohorts of patient tissues combined with genome editing technology and the development of new mouse models will likely shed more insights to the roles of PKD in health and diseases. The information will also help to design more effective and less toxic therapeutic strategies and agents to target PKD for the treatment of diseases.

## Figures and Tables

**Table 1:** Contributary roles of PKD isoforms in cancer.

Pathological Events	PKDs	Proposed Function	Cancer Types	Target Gene/Mechanisms	Ref
Proliferation	PKD1	Positive	- Pancreatic	- Stimulates accumulation of c-Fos, DNA synthesis via strengthening ERK while suppressing JNK/c-Jun signaling	[51]
				- Drives the formation of acinar-to-ductal metaplasia and further progression to pancreatic intraepithelial neoplasia	[52]
				- Prolongs ERK1/2 activation	[53,54]
				- Cell cycle regulation	[55,56]
			- Head and neck squamous cell; Kidney	- MEK/ERK-dependent signaling pathway	[57]
			- Prostate	- Increases ERα expression and cell sensitivity to 17β-estradiol	[58]
			- Breast	- Contributes to hyperplastic and inflammatory responses to topical phorbol ester	[41]
		Negative	- Prostate	- Increases MMP-2, MMP-9 secretion	[59]
				- Induces G1-phase arrest by phosphorylating cell-division cycle phosphatase 25	[60]
			- Colon	- Induces nuclear exclusion of β-catenin	[61]
			- Lung	- Negative regulator of mTORC1-S6K1 signalling	[62]
	PKD2	Positive	- Prostate	- Activated during G2-M, co-localizes with/regulate Aurora A kinase at the centrosome	[63]
			- Colon	- Stimulates NF-κB activity via AKT and ERK signalling	[64]
			- Glioblastoma	- Regulates Golgi phosphoprotein 3	[65]
	PKD3	Positive	- Breast	- Regulates mTORC1-S6 kinase 1 signalling	[66]
				- Activates ERK1/c-Myc axis	[67]
				- Phosphorylates HSP27 and HDAC4/5/7	[68]
Survival	PKD1	Positive	- Pancreatic	- Activates glucose transporter 1 and mTORC1	[69]
				- Induces anti-apoptotic proteins survivin and c-FLIP_L_	[70]
			- Prostate	- Activates ERK1/2 and NF-κB signalling	[8]
	PKD2	Positive	- Prostate; Colon; Leukemia	- Stimulates NF-κB activity	[8,71,72]
			- Colon; Breast	- Reverses HSP90 inhibition-induced apoptotic effects	[45]
	PKD3	Positive	- Prostate	- Akt and ERK1/2	[73]
EMT Migration Invasion	PKD1	Negative	- Prostate; Breast	- Inactivates transcription factor Snail	[74,75]
			- Prostate	- Phosphorylates junctional proteins (E-cadherin and β-catenin)	[76,77]
				- Polyubiquitination and proteasomal degradation of MTA1	[78]
			- Breast	- Promotes ɑvβ3 integrin recycling via phosphorylating Rabaptin-5	[79]
				- Represses the expression of MMPs	[80]
			- Melanoma	- Phosphorylates SSH1L, block cofilin dephosphorylation	[81]
				- Regulates E-cadherin expression and β-catenin localization	[82]
	PKD2	Positive	- Prostate	- Phosphorylates IKKβ, nuclear translocation and activation of NFκB	[83]
			- Pancreatic- Glioblastoma	- Stimulates expression and secretion of MMP-7 and MMP-9	[84]
			- Liver	- Regulates MMP-1 and integrin expression	[85]
			- Prostate	- Promotes PI3K/Akt/GSK-3β signalling	[86]
	PKD3	Positive	- Breast	- Activates NFκB and deactivate HDAC1	[83]
				- Secretion of MMP-9 and tumor-promoting cytokines	[87]
				- Activates PAK4/LIMK signaling	[88]
				- Regulates cytoskeletal remodeling by phosphorylating GIT1	[89]
Angiogenesis	PKD1	Positive	- Pancreatic	- Induces the secretion of VEGF and CXC chemokines	[90]
			- Breast	- LPA/PKD-1-CD36 signaling	[91]
	PKD2	Positive	- Gastrointestinal	- Regulates tumor-endothelial cell communication	[92]
			- Colon; Breast	- Stabilizes Hsp90; NF-κB/VEGF-A	[44,45]
	PKD3	Positive	- Prostate	- Regulates mast cell recruitment	[47]
Immune response	PKD2	Positive	- Oral squamous	- Regulates PD-L1 surface expression	[93]
			- Fibrosarcoma	- Phosphorylates and degrades IFNAR1	[94]
	PKD3	Positive	- Oral squamous cell carcinoma	- Regulates IFN-γ induced PD-L1 expression	[50]

**Table 2:** Versatile roles of PKD in human diseases besides cancer.

Disease	PKDs	Functions	Diseases/Pathologies	Targets	Ref
Cardiovascular disease	PKD1	- PKD1 activation leads to cardiac hypertrophy.	- Cardiac hypertrophy	- HDAC4, 5, 7, 9; MEF2	[146]
				- AKT/mTOR regulated autophagy	[144]
		- Regulates VEGF-mediated angiogenesis.	- Vasodilation	- Nitric oxide synthase	[148]
	PKD3	- Mediates glucose uptake during cardiac hypertrophy.	- Cardiac hypertrophy	- NFATc4, Nkx2.5, GATA4, MEF2	[149]
CNS disorders	PKD1	- Maintains polarity of hippocampal neurons	- Neuronal polarization and development	- Kidins220, Par-1	[153,154,155]
		- Neuronal survival	- Neurodegeneration	- NF-κB	[161]
			- Ischemic stroke	- Hsp27	[162]
		- Mediates neurogenic inflammation and pain transmission	- Hyperalgesia	- TRPV	[184]
	PKD2	- Maintains neuronal polarity	- Neuronal polarization and development	- Kidins220	[154]
		- Contributes to autism spectrum disorder	- ASD, RTT	- ERK1/2	[156]
		- Mediates neurogenic inflammation and pain transmission	- Hyperalgesia	- TRPV	[184]
	PKD3	- Expressed in primary sensory neurons that mediate neurogenic inflammation and pain transmission	- Hyperalgesia	- TRPV	[184]
Metabolic disease	PKD1	- Regulates insulin secretion and pancreatic β cell survival; Insulin exocytosis at TGN	- Type 2 diabetes, obesity	- Inhibitory phosphorylation by p38δ	[24]
	PKD2	- PKD2 inhibition leads to insulin resistance	- Hyperinsulinemia	- L-type Ca^2+^ channels	[166]
	PKD3	- Suppresses insulin signalling in liver and promotes insulin resistance	- Type 2 diabetes	- Akt/mTORC1 and mTORC2	[168]
Inflammatory disease	PKD1	- Contributes to bacteria-induced proinflammatory immune responses and neutrophil influx	- Hypersensitivity pneumonitis	- MAPK, NF-κB	[169]
		- Inflammatory cell infiltration	- Pancreatitis	- NF-κB, IL-6, MCP-1	[179]
		- Contributes to fibrosis	- Fibrosis	- HDACs, MEF2	[146]
	PKD3	- Liver fibrosis, hepatic macrophage polarization	- Liver fibrosis	- TGFβ	[187]
Immune dysregulation	PKD1	- Mast cell activation	- Allergic reaction	- MCP-1	[191]
		- Activated by TLR ligands, and is MyD88-dependent	- Proinflammatory immune responses	- TRAF6, TAK1, MAPKs	[192]
		- Transcriptional activates Nur77 during thymocyte activation	- T-cell receptor activation	- HDAC7	[195]
	PKD2	- Excessive cell autonomous T follicular helper cell development	- Germinal center development	- Bcl6	[200]
		- Nature killer cell activation	- Innate immune response	- IFN-γ, TNF-α	[48]

## Data Availability

All data and materials are available and support the published claims and comply with field standards.

## Abbreviations

PKD	protein kinase D
DAG	diacylglycerol
CNS	central nervous system
CAMK	Ca^2+^/calmodulin-dependent protein kinase
GPCR	G protein-coupled receptor
PH	pleckstrin homology
ULD	ubiquitin-like domain
PLC	phospholipase C
PKC	protein kinase C
TGN	trans-Golgi network
ROS	reactive oxygen species
ERK	extracellular-regulated protein kinase
NF-κB	nuclear factor-κB
mTOR	mammalian target of rapamycin
HDAC	histone deacetylase
MMP	matrix metalloproteinase
EMT	epithelial-to-mesenchymal transition
SSH1L	slingshot protein phosphatase 1L
LIMK1	LIM domain kinase 1
MTA1	metastasis-associated protein 1
ECM	extracellular matrix
VEGF	vascular endothelial growth factor
MEF2	myocyte enhancer factor 2
